# Dietary Management, Clinical Status and Outcome of Patients with Citrin Deficiency in the UK

**DOI:** 10.3390/nu12113313

**Published:** 2020-10-29

**Authors:** Alex Pinto, Catherine Ashmore, Spyros Batzios, Anne Daly, Charlotte Dawson, Marjorie Dixon, Sharon Evans, Diane Green, Joanna Gribben, Inderdip Hunjan, Elisabeth Jameson, Camille Newby, Germaine Pierre, Sanjay Rajwal, Louise Robertson, Si Santra, Mark Sharrard, Roshni Vara, Lucy White, Gisela Wilcox, Ozlem Yilmaz, Anita MacDonald

**Affiliations:** 1 Birmingham Women’s and Children’s Hospital, Birmingham B4 6NH, UK; catherine.ashmore@nhs.net (C.A.); a.daly3@nhs.net (A.D.); evanss21@me.com (S.E.); s.santra@nhs.net (S.S.); o.yilmaz@ybu.edu.tr (O.Y.); anita.macdonald@nhs.net (A.M.); 2 Great Ormond Street Hospital for children, NHS Foundation Trust, London WC1N 3JH, UK; spyros.batzios@gosh.nhs.uk (S.B.); marjorie.dixon@gosh.nhs.uk (M.D.); 3 University Hospitals Birmingham NHS Foundation Trust, Birmingham B15 2TH, UK; charlotte.dawson@uhb.nhs.uk (C.D.); louise.robertson@uhb.nhs.uk (L.R.); 4 Salford Royal NHS Foundation Trust, Manchester M6 8HD, UK; diane.green@srft.nhs.uk (D.G.); gisela.wilcox@srft.nhs.uk (G.W.); 5 Evelina London Children’s Hospital, London SE1 7EH, UK; joanna.eardley@gstt.nhs.uk (J.G.); roshni.vara@gstt.nhs.uk (R.V.); 6 Bradford Teaching Hospitals NHS Foundation Trust, Bradford BD9 6RJ, UK; inderdip.hunjan@bthft.nhs.uk; 7 Manchester University NHS Foundation Trust, Manchester M13 9WL, UK; elisabeth@doctors.org.uk; 8 Bristol Royal Hospital for Children, Bristol BS2 8BJ, UK; camille.newby@uhbw.nhs.uk (C.N.); germaine.pierre@uhbw.nhs.uk (G.P.); 9 Leeds Children’s Hospital, Leeds LS1 3EX, UK; sanjay.rajwal@nhs.net; 10 Sheffield Children’s NHS Foundation Trust, Sheffield S10 2TH, UK; mark.sharrard@nhs.net (M.S.); lucy.mulrooney@sch.nhs.uk (L.W.); 11 School of Medical Sciences, Faculty of Biology Medicine & Health, University of Manchester, Manchester M13 9PL, UK; 12 Department of Nutrition and Dietetics, Ankara Yildirim Beyazit University, Ankara 06760, Turkey

**Keywords:** citrin deficiency, NICCD, FTTDCD, outcomes, clinical status, dietary management

## Abstract

Background: Little is known about the optimal dietary treatment for citrin deficiency. Our aim is to describe the management of UK citrin deficiency patients. Methods: A longitudinal retrospective review was performed. Data were collected from medical records on presenting signs and symptoms, dietary management and clinical outcome. Results: data were collected on 32 patients from 21 families. 50% were females (16/32). Median age at diagnosis was 4 y (5 days–35 y) with 12 patients diagnosed in the neonatal period with neonatal intrahepatic cholestasis (NICCD), eight later in childhood (FTTDCD) and 12 by family screening based on index cases from five families. No patient had adult-onset type II citrullinemia. The patient age at the time of data collection was a median of 11 y (1–44 y). 91% (29/32) of patients had normal physical and neurological development, 47% (15/32) experienced recurrent unexplained abdominal pain and 9% (3/32) episodes of hypoglycaemia. Siblings had different phenotypes (5 families had > 1 affected patient). Most patients preferred high protein foods, limiting sugar-containing foods. Only 41% (13/32) were prescribed a low CHO, high protein, high fat diet (restriction varied) and two used medium chain triglyceride (MCT) supplements. No patient was prescribed drug therapy. Twenty-five per cent (8/32) of patients were underweight and 41% (13/32) had height <−1 z-scores. Conclusions: patients presented with various phenotypes, symptoms and suboptimal growth. Symptoms and biochemical markers improved with age, but height remained low in some. More research is necessary to assess the effectiveness of dietary approaches in improving clinical outcomes and symptoms in citrin deficiency.

## 1. Introduction

Citrin deficiency is a complex, rare, autosomal recessive disorder due to mutations in the SLC25A13 gene. In the 1950s, the adult disorder, citrullinemia type II (CTLN2), was first described, but it was not until the late 1990s that a Japanese team identified the citrin gene [1]. Citrin deficiency is mostly reported in Asia, but it is also detected in other countries [2]. Citrin is an aspartate/glutamate carrier and part of the mitochondrial malate-aspartate nicotinamide adenine dinucleotide hydrogen (NADH) shuttle. Its deficiency has several metabolic effects including: a decrease in the transport of aspartate from the mitochondria into the cytoplasm causing urea cycle disruption; inhibiting NADH supply to the mitochondria decreasing adenosine triphosphate (ATP) production [3]; inhibition of gluconeogenesis from lactate and glycerol due to the disturbance in NADH-NAD^+^ balance; and impact in protein and nucleotide synthesis [3]. Fatty acids are a major source of energy for hepatocytes and an energy deficit is caused by impaired hepatic glycolysis and de novo lipogenesis [4].

Given the important and multiple roles of citrin, its deficiency can manifest with a wide range of symptoms [5] and its clinical characteristics vary with age. There are three main phenotypes: (1) neonatal intrahepatic cholestasis (NICCD), which usually resolves by 1 year of age; (2) failure to thrive and dyslipidemia caused by citrin deficiency (FTTDCD) with common presenting symptoms including ketotic hypoglycaemia, dyslipidaemia, lethargy, hyperlipidaemia, pancreatitis and/or hepatoma in children aged 1–11 years old; and (3) adult-onset citrullinemia type 2 (CTLN2) in adolescents and adults (11–79 years old) [5]. Patients with CTLN2 may develop neuropsychiatric symptoms, generally associated with hyperammonaemia, liver dysfunction and growth deficiencies. In CTLN2, carbohydrate (CHO) toxicity associated with brain oedema and coma is also described [5].

The primary treatment of citrin deficiency is diet therapy. Medium-chain triglycerides (MCT) added to infant formula/breast milk is advocated for infants with NICCD to improve cholestasis. A low lactose formula may also be recommended in cases of hypergalactosemia [3,6,7]. A high protein (range 15 to 20% of energy), high fat (range 40 to 50% of energy) and low CHO diet (range 40 to 50% of energy) is advocated [3] for patients >1 year. Energy intake should meet estimated average requirements for age.

Reports suggest that patients have aversions to high CHO foods and prefer higher protein/fat foods. A high CHO (including complex sources) intake is not recommended as it causes toxicity [2] by increasing NADH in the hepatocyte cytoplasm, which inhibits glyceraldehyde 3-phosphate dehydrogenase [8]. This leads to disturbances in urea synthesis, and stimulates the citrate-malate shuttle, which may cause high triglyceride levels, high ammonia and/or fatty liver. When considering glucose metabolism, hepatic glucose uptake and metabolism are limited under normoglycaemia, owing to the low-glucose affinity of glucose transporter 2 (GLUT2) in hepatocytes [7,9]. However, in a case report describing two patients with citrin deficiency and diabetes mellitus type 2 with persistent hyperglycaemia, glucose was toxic; it was suggested that glucose uptake was increased leading to metabolite accumulation, ATP depletion and hepatocyte damage [7,9]. Protein and fat are used as alternative energy sources, and in a mouse model, it has been proposed that a high protein intake and alanine supplementation may increase CHO tolerance, improving appetite and weight gain [5]. It is also known that amino acids and fatty acids produce ATP independently of the NADH shuttle [9], which may explain patient preference.

Dietary practices and prescriptions are not well described in citrin deficiency. In UK patients, we aimed to characterise differences in dietary prescriptions compared with patients’ outcome and biochemical and clinical status to help direct future research in the dietary treatment of citrin deficiency.

## 2. Materials and Methods

### 2.1. Study Design and Subjects

A multicentre longitudinal, retrospective case record review of patients diagnosed with citrin deficiency from 10 UK metabolic centres was completed. A research dietitian from Birmingham Women’s and Children’s Hospital (A.P.) collected data from case records of patients throughout the UK. All the UK centres caring for patients with citrin deficiency were identified through the British Inherited Metabolic Diseases (BIMDG) Dietitians Group and then invited to participate. The following centres contributed patients: Birmingham Women’s and Children’s Hospital (*n* = 7); University Hospitals Birmingham NHS foundation trust (*n* = 1); Bristol Royal Hospital for Children (*n* = 1); Guy’s and St Thomas’ NHS foundation Trust, London (*n* = 4); Evelina Children’s Hospital, London (*n* = 6) Great Ormond Street Hospital for Children NHS Foundation Trust, London (*n* = 6); Salford Royal NHS Foundation Trust, Manchester (n=1); Bradford Teaching Hospitals NHS Foundation Trust (*n* = 2); The Leeds Teaching Hospitals NHS Trust (*n* = 3); and Sheffield Children’s NHS Foundation Trust (*n* = 1).

### 2.2. Inclusion Criteria

Any patients diagnosed with proven citrin deficiency based on biochemistry and/or genetics. This included patients with NICCD, FTTDCD and CTLN2. All patient ages were included.

### 2.3. Exclusion Criteria

Citrin deficiency unconfirmed by diagnostic procedures or patients unable or unwilling to give consent.

### 2.4. Data Collection

The following information was collected from medical and dietetic records: clinical manifestations at presentation, age at diagnosis, diagnosis/genetic confirmation, parental consanguinity and ethnic origin, family history, siblings with citrin deficiency, medical history, hospital admissions, length of hospital stay with reasons for admissions and emergency department attendances with purpose of visit. Lifetime dietary prescriptions, type and severity of dietary restrictions and median % energy intake from protein, CHO and fat were recorded. Any prescription for MCT oil, MCT infant formula, vitamins, minerals or any other dietary supplementation, nutritional support (tube feeding, e.g., nasogastric tube or gastrostomy tube feeding), and emergency feeding plans were documented. Food preferences, dietary adherence, medications/drug therapy and growth (weight and height) were recorded. Data on psychomotor development, neuropsychiatric symptoms, co-morbidities, medical outcome and clinical status, biochemical markers (nutritional biochemical markers and quantitative amino acids) and results of any other investigations were collected.

### 2.5. Dietary Intake

Dietary intake was calculated from retrospective food histories (24 hour recall/3 day diet history), using the Nutritics^®^ program [10]. When more than one dietary assessment was available, data were presented as a median.

### 2.6. Growth

Data were collected on height and weight and converted to age based z-scores for weight and height and body mass index (BMI) according to WHO/UK growth definitions [11]. We have used different margins to characterise non-standard growth in our cohort. Underweight was defined according to weight-for-age z-score [12] and stunting according to height-for-age z-scores [12]. Overweight and obesity were defined by BMI-for-age z-scores (5–19 years) or by BMI (>19 years of age) [13,14]. The definitions used are described in Table 1.

### 2.7. Ethical Statement

National favourable ethical opinion by the West Midlands—Solihull research ethics committee (reference 17/WM/0410 and IRAS 2333728) was given to conduct this study. All eligible patients or carers of children were identified and sent a study information sheet. When further information was requested by the patients or carers, clinicians from the local health team discussed the research with them. Parents or patients aged over 16 years gave written informed consent and children assented when applicable (dependent on age and understanding).

### 2.8. Statisticsa

Descriptive statistics was used to analyse data (percentages, medians and ranges).

## 3. Results

### 3.1. Subjects

Ten of 12 UK centres with known cases of citrin deficiency participated in this study. Thirty-two of 35 known UK patients were recruited, with data collected on all 32 patients (50% females). Almost all patients (*n* = 30) were of Asian origin (mostly Pakistani), except two patients of white British origin. Fifty-six per cent (18/32) were from consanguineous families; in total 15/32 patients were from five families. Ninety-three per cent (*n* = 14/15 patients with available data) were born full term with a median birth weight (*n* = 12) of 2.8 kg (range 2.48 to 3.26 kg).

Overall, the median age at diagnosis was 4 y (5 days to 35 y), with 12 presenting clinically in the neonatal period (NICCD), eight later in life (we used FTTDCD to define patients presenting later in life during the adaptation/compensation stage irrespective of presentation) and 12 by sibling/family screening. 

Median age of the sibling/family screening was 7 y (1–35 y). No patient had CTLN2. In comparison, median age of presentation when data available was 2.5 y (1–12 y). Median patient age data collection was 11 y (1–44 y). Eight patients had co-existing disorders not related to citrin deficiency (glycogen storage disease type IX [*n* = 1], coeliac disease [*n* = 3 from two families], asthma [*n* = 1], congenital hypothyroidism [*n* = 2] and hypoplastic left heart syndrome (HLHS) [*n* = 1]).

### 3.2. Signs and Symptoms at Presentation

Patients varied widely in presentation, descriptions included: neonatal cholestasis (*n* = 8), jaundice (*n* = 10), hepatitis (*n* = 3), elevated phenylalanine (Phe) and tyrosine (Tyr) (*n* = 4), abnormal plasma amino acids with raised citrulline (*n* = 8), raised threonine (*n* = 2), raised methionine (*n* = 1) hypoglycaemia (*n* = 6), galactosuria (*n* = 2), hyperammonaemia (*n* = 2), abnormal lactate (*n* = 2), febrile seizures (*n* = 1), pale stools (*n* = 1) and faltering growth (*n* = 1).

At the time of data collection, 91% of patients (*n* = 29/32) had normal physical ability and neurological development. Three were reported to have either bowed legs, which later improved (*n* = 1), reduced leg strength (*n* = 1) and some speech delay (*n* = 1). Six patients had symptoms of ketotic hypoglycaemia following diagnosis. Forty-seven per cent (*n* = 15/32) had reported recurrent abdominal symptoms with or without hypoglycaemia. Only one patient presented with abdominal pain due to constipation; the remaining subjects had no documented cause for the abdominal pain.

### 3.3. Diagnosis and Biochemistry

All data on patients’ characteristics, demographics and presentation are presented for NICCD (Table 2), FTTDCD (Table 3) and family screened patients (Table 4).

### 3.4. Dietary Treatment

Dietary management and food preferences together with growth outcomes are presented for all patients in Table 5.

### 3.5. Diet Prescriptions

Only 41% (*n* = 13/32) of patients were prescribed a dietary restriction (Table 5). Eight of 32 patients (25%) were given a low CHO, high protein and high fat diet; two patients were prescribed a low CHO, high protein and high fat diet supplemented with MCT. One patient with ongoing severe symptoms (abdominal pain/sweatiness) was prescribed a very low CHO (30 g/day), high protein and fat diet (similar to an Atkins diet), supplemented with MCT/ long chain triglyceride (LCT). Five patients were prescribed and two continued a hydrolysed casein formula (55% of total fat from MCT) following neonatal presentation. One patient was given soya formula during infancy.

From 27 patients that had available data about their dietary intake, 16 were documented to follow a normal diet. However, all appeared to self-restrict their dietary intake as shown in Table 5.

### 3.6. Vitamin and Mineral Supplements and Tube Feeding

Eight of 32 patients (25%) were prescribed vitamin and mineral supplements due to limited intake. One symptomatic patient received home enteral feeding to help control symptoms (extreme abdominal pain and sweating). This was initially administered via a nasogastric tube but later replaced by gastrostomy feeding. This patient had several hospital admissions due to extreme abdominal pain.

### 3.7. Dietary Intake

Twenty-seven of 32 (84%) patients had dietary intake data documented, either by 24 h recalls or 3-day food records. The median percentage (range) of energy from CHO, protein and fat is given in Table 6. Values are shown in total and for each different presentation of the disorder.

With or without formal dietary advice and including patients on a “normal diet”, most patients followed a low CHO, high protein and high fat diet, similar to that recommended by Okano et al. in 2019 [3] (30–40% energy from CHO, 15–25% energy from protein and 40–50% energy from fat). Compared with recommendations for the general population (50–60% energy from CHO, 10–15% energy from protein and 25–35% energy from fat) [3], this patient cohort had a lower CHO but higher protein and fat intake.

Patients not prescribed diet therapy had a higher percentage of energy intake from CHO and lower percentage of energy from fat compared with patients on diet (Table 7). However, they still had a lower CHO, higher protein and higher fat intake when compared to the recommended diet for a healthy population.

Patients had an estimated median energy intake of 86% (range 22–123%) compared with estimated average requirements for age [15].

### 3.8. Food Preferences

Most patients preferred high protein foods (meat, fish, cheese, nuts, cow’s milk) and avoided high CHO foods especially sweets and sugary drinks at the last assessment (median age 11 y). Some patients chose higher CHO foods in early childhood, but dietary records indicated avoidance later (Table 5).

### 3.9. Emergency Regimens

Twenty of 32 patients (from eight of 10 centres) were prescribed an emergency regimen to help control/prevent symptoms during illness (Table 5). Emergency regimens varied widely in composition: full fat milk only (*n* = 11/32), hydrolysed casein formula with MCT (*n* = 2/32), soya milk (*n* = 3/32), unsweetened soya milk (*n* = 1/32), unsweetened soya protein drink with LCT/MCT emulsion (*n* = 1/32) and low sugar soya milk with MCT emulsion with added soya protein (*n* = 1/32) (Table 5).

During hospital admissions, IV management followed the BIMDG guidelines for citrin deficiency [16].

### 3.10. Hospital Admissions

The median number of hospital admissions was one (1–4) prior to diagnosis and one (1–8) post-diagnosis. Only three patients had > 3 hospital admissions. Admissions were mainly associated with hypoglycaemia and severe abdominal pain. 

### 3.11. Drug Therapy

No patient was prescribed sodium pyruvate at the time of data collection. One patient was given Movicol^®^ for constipation and one Ranitidine^®^ for gastroesophageal reflux.

### 3.12. Growth

**Figure 1:**
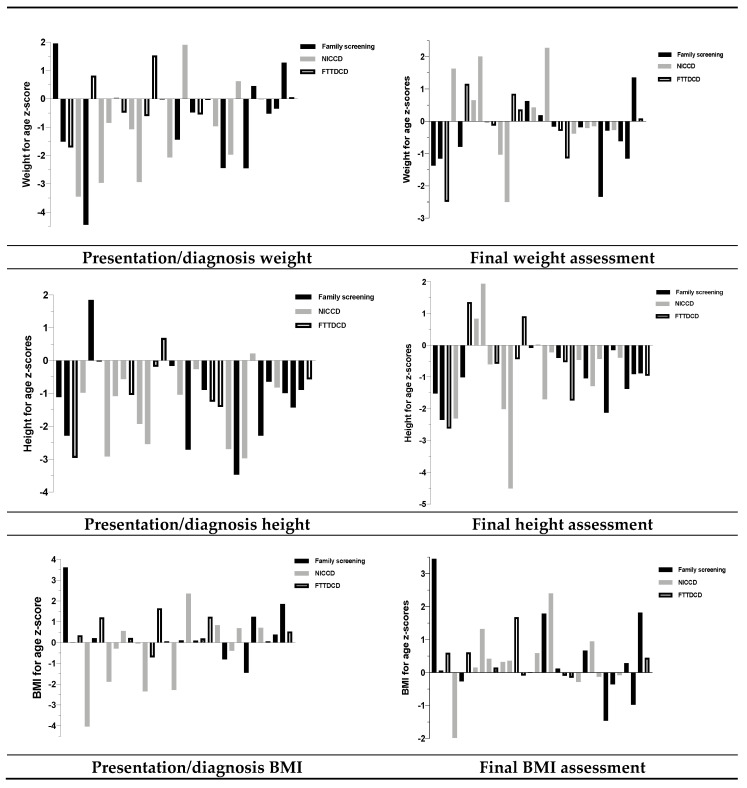
Presentation/diagnosis and final weight-for-age, height-for-age and BMI-for-age z-scores for each individual patient. Abbreviations: BMI: Body mass index; NICCD—neonatal intrahepatic cholestasis; FTTDCD—failure to thrive and dyslipidaemia caused by citrin deficiency.

Weight, height and BMI for age z-scores are presented for each individual patient at presentation/diagnosis and last assessment in Figure 1. Type of presentation/diagnosis (NICCD, FTTDCD and family screening) is given.

In general, at presentation/diagnosis, 28% (9/32) had height z-score < −2 and 50% (16/32) < −1. Height z score improved in some patients, but 41% (13/32) remained < −1 and 19% (6/32) < −2 height z score (Table 1 and Table 5).

Fifty-four per cent (7/13) of patients with low height z score were symptomatic with recurrent abdominal and/or hypoglycaemia. Forty-six per cent (6/13) were prescribed a low CHO, high fat, low sugar diet.

Thirty-eight per cent of patients (*n* = 12/32) were underweight at presentation/diagnosis and 25% (*n* = 8/32) at the final evaluation. Additionally, at the final evaluation, 19% (6/32) were overweight/obese; two of seven adults and four of 25 children.

### 3.13. Biochemical Markers

**Figure 2:**
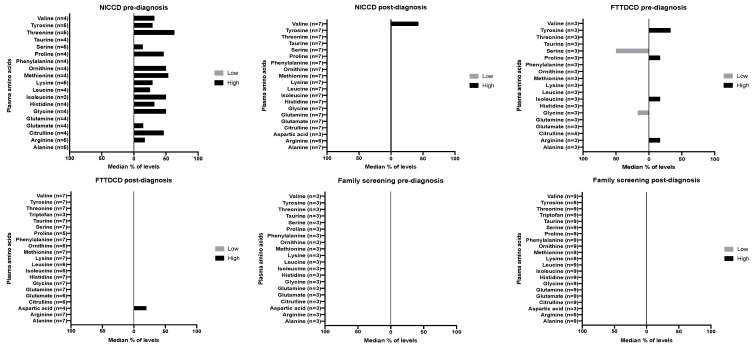
Median % of levels > and < reference range for plasma amino acids levels pre- and post-diagnosis. Abbreviations: NICCD—neonatal intrahepatic cholestasis; FTTDCD—failure to thrive and dyslipidemia caused by citrin deficiency; N—number of patients with each biochemical measure. A minimum of three patients results were included for amino acids. Normal reference ranges were specific for each laboratory and patient’s age.

Data on plasma amino acids are given in Figure 2. This is presented as the median percentage of amino acid levels > or < reference range. Target ranges differed between different centres, age groups and even in the same laboratory throughout the years. Data on each specific amino acid were only presented if there was data for at least three patients. Data are given for pre- and post-diagnosis in each of the presentation/diagnosis groups (NICCD, FTTDCD and family screening).

**Figure 3:**
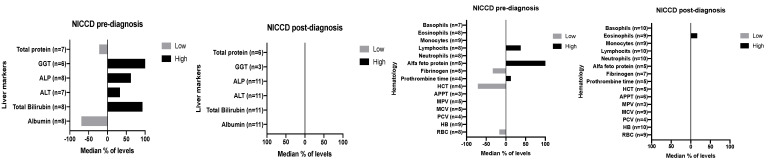
Median % of levels > and < reference range for liver biochemical markers and haematology parameters pre- and post-diagnosis in NICCD patients. Abbreviations: NICCD—neonatal intrahepatic cholestasis; ALT—Alanine aminotransferase; ALP—alkaline phosphatase; GGT—gamma-glutamyl transferase; RBC—red blood cell; HB—haemoglobin; PCV—packed cell volume; MCV—mean corpuscular volume; MPV—mean platelet volume; APTT—activated partial thromboplastin time; HCT—haematocrit; N—number of patients with biochemical measurement. A minimum of three patients results were included for each biochemical marker. Normal reference ranges were specific for each laboratory and patient age.

Haematology parameters and liver biomarkers are presented in Figure 3. A median percentage of levels > or < reference range is given for NICCD patients. There was inconsistency in the type of markers analysed, varying widely across centres. Data were only presented if they were available for at least three patients. Liver markers remained within normal range for FTTDCD and family screening patients pre- and post-diagnosis. For haematology markers, the median percentage of levels were within normal range, except for lower levels of haematocrit (in FTTDCD pre-diagnosis) and mean corpuscular volume (in FTTDCD post-diagnosis). In the family screening group, most markers were within range, except for lower levels of packed cell volume, haemoglobin and red blood cell pre- and post-diagnosis. Patients had limited biochemical data for lipid metabolism (e.g., cholesterol, triglycerides) and renal markers.

## 4. Discussion

This study is the largest cohort of patients with citrin deficiency reported outside of Asia; previously there have only been small numbers of case reports described in non-Asian countries [2]. Our cohort had variable phenotypes, even within the same family. The prescribed treatment for citrin deficiency was a low CHO, high protein, high fat diet, which was individualised according to patient’s symptoms. A specific percentage of energy from CHO, protein and fat was not usually given, although patients commonly consumed comparable amounts to the Okano et al. recommendations [3] and other previous reports [17]. Patients given a prescribed diet had similar percentage energy contributions from CHO, protein and fats to patients without formal dietary restriction. Food aversions were common, with most patients preferring high protein foods compared to high CHO foods, and aversions were greater with increasing age.

It was unclear why some patients were prescribed low CHO, high protein, high fat diets and others not; this appeared independent of type or age of diagnosis. However, patients who were symptomatic post-diagnosis were more likely to be prescribed diet therapy. Each centre did not have the same approach for all patients. Some patients had already adapted their own dietary habits. It was unknown if further adjustment of protein, fat and CHO intake would have relieved some patients with ongoing symptoms. It is important to consider dietary treatment for all patients, as CHO restriction may help prevent disorder progression, and there are no reports describing patients with CTLN2 who started dietary treatment earlier in life [18,19]. There is a clear lack of guidance as to the optimal dietary management for patients with different presentations leading to different practices across the UK centres.

In our cohort, only two patients were given MCT supplements. Additionally, two young patients were given MCT supplemented casein hydrolysate formula in infancy, which continued beyond one year. MCT provides direct energy to the hepatic cells by supplying acetyl-CoA, promoting lipogenesis and increasing the cytosol NAD^+^/NADH ratio [9]. The effects of MCT on NICCD were originally described by Hayasaka et al. [7], and a single case reported by Otsuka et al. [20] suggested MCT therapy can prevent the onset of hypoglycaemia when patients are well. It is also suggested that infant formula with a higher MCT content or a normal infant formula supplemented with MCT may help resolve neonatal cholestasis [3]. In adults with CTLN2, it is thought that long-term MCT supplementation may help lower high ammonia and citrullinemia concentrations [7,9]. In our cohort, ammonia was usually monitored during hospital admissions only.

The use of arginine has previously been reported as an adjunct treatment in citrin deficiency [21]. It was suggested that arginine supplementation could facilitate ammonia detoxification by acting as a substrate for mitochondrial urea cycle enzymes [22,23]. Protein containing foods provide arginine, but foods such as nuts, seeds, soya protein and meat are particularly rich sources, and these were recommended by some centres to increase arginine intake. However, its precise mechanism is unknown, and the efficacy of arginine supplementation is not proven in citrin deficiency. Recently, it has been reported that ornithine plus aspartate significantly reduces blood ammonia in a mouse model of citrin deficiency [24] and soya protein and fish are also rich sources of aspartate.

Sodium pyruvate, which may be used in combination with MCT, has also been considered as a treatment option. In our cohort, no patients were prescribed sodium pyruvate even though this has been shown to improve growth and biochemical markers as it oxidises cytosolic NADH to NAD^+^ and provides energy to the tricarboxylic acid cycle [3] in patients with citrin deficiency [8]. Yazaki et al. treated 15 CTLN2 patients with sodium pyruvate on a low CHO diet and found a decrease in the frequency of hyperammonaemic encephalopathy in 11 patients. However, the treatment did not prevent relapse of encephalopathy or improve the Fischer ratio or citrullinemia [7].

A high CHO diet is unfavourable in citrin deficiency and it may trigger the onset of CTLN2. Thereby, during intercurrent infections to prevent metabolic decompensation, an alternative emergency regimen is advocated to the usual high intake of glucose supplied by glucose polymer drinks or IV dextrose given in other metabolic disorders [25]. In our cohort, different types of low CHO/high protein emergency drinks were prescribed to alleviate symptoms during illness. Some used full fat cow’s milk (*n* = 11) as the energy percentage composition is similar to the published self-selected diet prescribed [e.g., aim 100 ml cow’s milk = 65 kcals, protein 3.3 g (20%), CHO 4.7 g (29%), fat 3.8 g (52%)]. Six patients were given low sugar soya milk /soya protein. There are no data in our cohort to indicate how well tolerated, accepted or effective the different emergency drinks were in avoiding hospital admissions; this remains unreported in the literature. It is also unknown how long emergency drinks were continued for. Very few patients had repeated hospital admissions related to the disorder, suggesting that most could manage their illness at home.

Although there was some overall improvement in height following diagnosis or dietary management, suboptimal growth remained in some (*n* = 13). This was comparable to a previous report [4]. In one study [4], 111 NICCD-affected subjects and 12 NICCD unaffected subjects where studied. Both groups showed significant growth impairment, including low birth weight (similar to our cohort) and length. We present a mixed cohort of NICCD and FTTFCF, and there were no differences between the groups, except for the sibling-screened patients who, interestingly, had a higher percentage of lower heights. One possible explanation may be that these patients were diagnosed later in life (median age 11 y compared with the overall median age of presentation/diagnosis of 7 y). Moreover, this patient group was not prescribed stringent dietary treatment. In addition, many were of Asian origin, which could explain the findings. Patients appeared to have a low energy intake (median of 86% of EAR), and our results were similar to a Japanese study that reported a median energy intake of only 87% of recommended amounts [3]. As patients lower their CHO intake, it is important that any energy deficit is replaced by eating extra fat and protein or some energy sources such as MCT oil. Some suggest MCT is potentially beneficial, but more evidence is needed. It is also reported that excess CHO intake can lead to weight loss or poor growth [3]. It is important to monitor energy intake and nutritional status carefully.

Overall, the clinical features of this patient cohort were largely mild and non-specific, particularly once neonatal cholestasis had resolved, with few clinical or biochemical abnormalities. However, 47% (*n* = 15/32) did report recurrent abdominal pain or had proven hypoglycaemia (a small number of patients monitored blood glucose at home), with symptoms persisting even with diet therapy. It has been suggested that hypoglycaemia may be associated with inadequate CHO intake and decreased hepatic glycogen storage related to energy deficit in hepatocytes [7]. Hayasaka et al. [7] suggested that to avoid hypoglycaemia, CHO intake should supply 100 g/day in patients > 1 year of age and 130g/day in patients aged ≥ 5 years. We consider that the CHO intake should be adapted according to individual needs. It is important that estimated average energy requirements are met and blood glucose monitoring considered with a very restricted CHO intake.

Okano et al. [3] has reported severe fatigue, abdominal pain, anorexia and weight loss in patients with FTTDCD. Impaired quality of life is also reported [26]. In one of our cases, abdominal pain was related to constipation, but in most cases recurrent abdominal pain was unexplained. There are reports of pancreatitis due to hypertriglyceridemia which may be a cause of recurrent abdominal pain in some patients [7]. Triglycerides were not routinely monitored in the UK, but lipase, amylase and triglycerides should be considered as part of routine monitoring. It is important to closely monitor and document symptoms in routine clinical practice, as there is much to learn about the natural history of this condition. Future studies assessing symptoms and quality of life are necessary.

In general, biochemical markers were largely within normal reference range following diagnosis. There were some cases of low haemoglobin and MCV. This is probably not diet-related, as the majority of patients were of Asian origin, where iron deficiency is common [27]. Even pre-diagnosis, only NICCD patients had plasma amino acids, liver and haematology markers outside normal range. Patients presenting with other types of presentation/diagnosis generally had blood levels within range pre- and post-diagnosis. There were no patients with CTLN2 in this cohort. Practice and frequency of biochemical monitoring varied across the UK, and there were limited data for triglyceride and cholesterol concentrations.

There were more monitoring data available for NICCD patients compared to FTTDCD or patients diagnosed by family screening. Careful monitoring and assessment of symptoms is necessary as citrin deficiency does not always follow a benign course [28]. It is important to gain national consensus about follow up and agree on a management protocol and standards of care for patients with citrin deficiency. Okano et al. recommended, during early childhood, that growth, blood count, general biochemistry, amino acids, blood sugar, cholesterol and ammonia should be monitored at least 3 monthly, and patients at school age monitored every 4 to 6 months [3]. 

There are several limitations to this study. Data were collected retrospectively, thereby affecting its accuracy and completeness. We did not study an age and gender matched, healthy control group. Future longitudinal, prospective case control studies comparing patients with healthy controls is important to understand the relevance of all the symptoms we observed. We accept that symptoms such as abdominal pain occur commonly in the general population, but this patient cohort displayed symptoms similar to a Japanese group of patients with citrin deficiency [3,26]. Monitoring was not uniform across and within centres. All patients had different disease severity (both related to symptoms and biochemical findings), which constrained data comparisons. Only limited diet histories were available, and energy intake may have been underestimated due to incomplete records. We were also unable to estimate expected height, as there were no available data on parental height.

## 5. Conclusions

Patients presented with different phenotypes and biochemical abnormalities. In general, growth was below average and recurrent abdominal pain and hypoglycaemia were common. Most patients self-selected a low CHO diet with a preference for high protein foods, irrespective of formal dietary prescription. Symptoms in some patients improved but growth remained suboptimal, possibly related to low energy intakes.

It is important for healthcare professionals to clearly define guidelines about the optimal routine care and service that should be provided to support patients with citrin deficiency. Delineating frequency of appointments, biochemical analysis, dietary assessments, anthropometry and appropriate tools to assess symptoms is important. A uniform approach across the country would also enable future research assessing different dietary approaches in relation to clinical outcomes/symptoms in order to determine the optimal treatment for patients with citrin deficiency.

## Figures and Tables

**Table 1:** Definitions to characterise weight-for-age, height-for-age and BMI-for-age in patients with citrin deficiency.

	Weight-for-Age z-Score	Height-for-Age z-Score	BMI-for-Age z-Score(5–19 Years)
**Definitions**	Marginally underweight: <−1 Moderately underweight: <−2 Severely underweight: <−3 [12]	Marginally stunted: <−1 Moderately stunted: <−2 Severely stunted: <−3 [12]	Overweight: >+1 (or BMI ≥ 25 *) Obesity: >+2 (or BMI ≥ 30 *) [13,14]

Abbreviations: BMI: Body mass index; * in patients >19 years of age.

**Table 2:** Demographics, genetic results and symptoms on presentation for NICCD patients.

Subject	Ethnicity	Consanguinity	Gender	Mutations	Co-existing Conditions	Full Term	Breast/Bottle Feeding on Presentation	Presentation Age	Diagnostic Age	Current Age	Biochemistry at Presentation/Diagnosis	Symptoms Post-Diagnosis
4	Asian	Yes	Female	c.1763G>A (p.Arg588Gln)	Coeliac disease	NA	Regular infant formula	1 month	2 months	11 years	Neonatal cholestasis, abnormal lactate.	Jaundice, abdominal pain due to constipation. Headache, lethargic morning leg pain.
7	White	Yes	Male	1465T>C (C489R)	-	Yes	Breastfeeding	2 months	1 year	14 years	Neonatal cholestasis. High citrulline, threonine and methionine.	Severe abdominal pain.
8	White	Yes	Male	c.1173T>G (P.Tyr391X)	Asthma	Yes	Regular infant formula	1 month	6 months	6 years	Neonatal intrahepatic cholestasis and conjugated hyperbilirubinemias.	Jaundice, pale stools.
9	Asian	No	Female	c.852_855delTATG p.(Met285Profs*2)	-	Yes	Soya infant formula	4 months	5 months	5 years	Cholestasis, high citrulline, galactosuria.	Hepatitis, jaundice, hypoglycaemia.
11	Asian	Yes	Male	-	-	Yes	Casein hydrolysate with medium chain triglyceride (MCT) infant formula	1 month	1 month	1 years	Increased phenylalanine and tyrosine on newborn screening.	Asymptomatic.
12	Asian	No	Male	c.1763 G>A P. (Arg 588GIn)	-	Yes	Casein hydrolysate with MCT infant formula	2 weeks	8 months	1 years	NA	Prolonged jaundice.
16	Asian	Yes	Male	c.1763 G>A (p.Arg588Gln)	-	Yes	Casein hydrolysate with MCT infant formula	1 month	8 months	6 years	Deranged liver function tests, clotting associated with positive reducing substances in urine and raised plasma citrulline.	Asymptomatic.
18	Asian	No	Male	c.1763G>A (p.Arg588Gln)	Hypoplastic left heart syndrome	NA	NA	1 month	8 years	20 years	Cholestasis.	Acute liver failure, hypoglycaemia (resolved with age). Abdominal pain, sweating on morning waking.
22	Asian	Yes	Female	c.1763G>A (p.Arg588Gln)	-	NA	Casein hydrolysate with MCT infant formula	1 week	7 months	10 years	Raised phenylalanine and tyrosine, neonatal jaundice.	Recurrent abdominal pain and headaches.
24	Asian	No	Male	c.852_855delTATG p.(Met285Profs*2)	-	NA	Breastfeeding	1 day	2 months	4 years	Neonatal cholestasis.	Asymptomatic
25	Asian	No	Male	c.848 G>T p. (Gly283 Val)	-	Yes	Breastfeeding	2 months	5 months	3 years	Increased citrulline.	Conjugated jaundice.
28	Asian	No	Male	c.1763G>A (p.Arg588Gln)	-	Yes	Casein hydrolysate with MCT infant formula	1 month	1 month	8 years	Neonatal cholestasis, elevated phenylalanine and tyrosine on newborn screening.	Conjugated jaundice, hypoglycaemia.

Abbreviations: NA—Not available; MCT—medium chain triglyceride.

**Table 3:** Demographics, genetic results and symptoms on presentation for FTTDCD patients.

Subject	Ethnicity	Consanguinity	Gender	Mutations	Co-existing Disorders	Presentation Age	Diagnostic Age	Current Age	Biochemistry at Presentation/Diagnosis	Symptoms Post-Diagnosis
3	Asian	Yes	Female	c.1763G>A (p.Arg588Gln)	Coeliac disease	1 year	6 years	8 years	NA	Abdominal pain and hypoketotic hypoglycaemia (sweating when eating sugar).
6	Asian	Yes	Male	1610-1612 delTA Gins AT	GSD type IX	2 years	2 years	8 years	NA	Night sweats.
10	Asian	No	Male	c.550C>T p. (Arg 184*)	-	11 years	12 years	15 years	Neonatal hepatitis, carnitine deficiency.	Conjugated jaundice, hypoglycaemia, febrile seizure.
13	Asian	Yes	Female	c.1766 C>T (p.Ser589 Phe)	-	1 year	8 years	15 years	NA	Prolonged jaundice, abdominal pain.
14	Asian	No	Female	c. 1763G> A (p.Arg588Gln)	-	3 years	3 years	14 years	NA	Abdominal pain.
20	Asian	No	Female	c.1781G>A (p.Gly594As)	Hypothyroidism	7 years	8 years	11 years	Decompensated cirrhosis and thyroid antibodies, elevated plasma citrulline.	Asymptomatic.
21	Asian	No	Male	R588Q	-	12 years	12 years	12 years	Neonatal hepatitis syndrome, raised citrulline, mildly elevated ammonia.	Asymptomatic.
32	Asian	No	Female	c.1763G> A (p.Arg588Gln)	-	1 year	14 years	22 years	Cirrhosis and steatosis. Failure to thrive and hypertonia with generalised aminoaciduria, galactosemia and echogenic enlarged liver.	Floppiness and rickets. Abdominal pain when fasting for Ramadan (unknown cause). Failure to thrive.

Abbreviations: NA—Not available; GSD—glycogen storage disease.

**Table 4:** Demographics, genetic results and symptoms on presentation for patients diagnosed by family screening.

Subject	Ethnicity	Consanguinity	Gender	Mutations	Co-existing Disorders	Diagnosis Age by Family Screening	Current Age	Biochemistry at Presentation/Diagnosis	Symptoms Post-Diagnosis
1	Asian	Yes	Male	c.1763G>A(p.Arg588Gln)	Coeliac disease	6 years	6 years	NA	Asymptomatic.
2	Asian	Yes	Female	c.1763G>A(p.Arg588Gln)	-	8 years	10 years	NA	Abdominal pain.
5	Asian	Yes	Female	c.1763G>A(p.Arg588Gln)	-	5 years	6 years	NA	Asymptomatic
15	Asian	No	Female	c.1763G>A(p.Arg588Gln)	-	8 years	19 years	NA	Long history of severe abdominal pain.
17	Asian	Yes	Female	c.1763G>A(p.Arg588Gln)	-	4 years	4 years	Elevated citrulline and threonine.	Hypoglycaemia, abdominal pain with reduced appetite.
19	Asian	No	Male	c.1781G>A(p.Gly594Asp)	Hypothyroidism	1 year	4 years	Cholestasis, raised ammonia and lactate, galactosuria, high citrulline.	Asymptomatic.
23	Asian	Yes	Male	c.1763G>A(p.Arg588Gln)	-	2 years	2 years	Raised phenylalanine and tyrosine.	Asymptomatic.
26	Asian	No	Female	c.1763G>A(p.Arg588Gln)	-	35 years	44 years	NA	Abdominal pain, feels nauseous with certain foods, abdominal cramps and frequent bowel movements.
27	Asian	Yes	Male	c.1763G>A(p.Arg588Gln)	-	5 years	13 years	NA	Asymptomatic.
29	Asian	Yes	Female	c.1763G>A(p.Arg588Gln)	-	9 years	19 years	NA	Abdominal pain.
30	Asian	Yes	Female	c.1763G>A(p.Arg588Gln)	-	15 years	22 years	NA	Abdominal pain.
31	Asian	Yes	Female	c.1763G>A(p.Arg588Gln)	-	12 years	20 years	Unconjugated jaundice.	Abdominal pain.

Abbreviations: NA—Not available.

**Table 5:** Dietary treatment, food preferences/aversions and growth parameters of all study participants.

Subject	Presentation	Growth at Presentation (Height-for-Age z-Scores)	Last Growth (Height-for-Age z-Scores)	Food Aversions/Preferences	Current Prescribed Dietary Management	Median Intake from Dietary Assessments	Oral Emergency Feed for Illness Management
1 (Family 1)	Family screening	−1.11	−1.52	Likes custard.	Normal diet.	CHO: 42% Protein: 18% Fat: 40%	Not prescribed.
2 (Family 1)	Family screening	−2.29	−2.35	Avoids: sweets, puddings, fruit juice, cakes, biscuits. Prefers: meat, fish, cheese, eggs, soya, nuts.	Low sugar, high protein, high fat diet.	CHO: 28% Protein: 20% Fat: 53%	Low sugar soya milk.
3 (Family 1)	FTTDCD	−2.96	−2.62	Avoids: sweets, puddings, fruit juice, cakes and biscuits. Prefers: meat, fish, cheese, eggs, soya, nuts.	Low sugar, high protein, high fat diet + MCT oil. One oral feed in the middle of the night.	CHO: 30% Protein: 19% Fat: 51%	Low sugar soya milk + MCT emulsion + soya protein powder.
4	NICCD	−0.98	−2.31	Initial self- restriction of sweets but now eats them (with symptoms). Prefers high protein foods.	Normal diet (but feeds 4 hourly day and night).	CHO: 44% Protein: 18% Fat: 43%	Not prescribed.
5 (Family 2)	Family screening	1.85	−1.01	Dislikes sweets.	Normal diet (but feeds 4 hourly day and night).	NA	Not prescribed.
6 (Family 2)	FTTDCD	−0.03	1.36	Dislikes sweets. Likes meat.	Low sugar, high protein, high fat diet (MCT).	NA	Liquid chocolate yoghurt.
7	NICCD	−2.91	0.84	Dislikes CHO foods, e.g., bread, rice, potatoes, sweets. Prefers high protein foods, e.g., bacon, eggs, nuts.	Low sugar, high protein high fat diet (LCT/MCT). CHO limited to 30g/day.	CHO: 18% Protein: 27% Fat: 55%	Soya protein powder + LCT/MCT emulsions + water.
8	NICCD	−1.08	1.94	None described.	Normal diet.	CHO: 47% Protein: 19% Fat: 33%	Not prescribed.
9	NICCD	−0.57	−0.60	Avoids sugar and likes cheese, fish, potatoes.	Normal diet.	CHO: 42% Protein: 23% Fat: 35%	Soya milk.
10	FTTDCD	−1.05	−0.58	Avoids sugar.	Normal diet.	CHO: 45% Protein: 19% Fat: 36%	Not prescribed.
11	NICCD	−0.43	−2.01	Eats sugary foods. Dislikes cheese.	Low sugar, high protein, high fat diet. Casein hydrolysed formula with MCT.	CHO: 40% Protein: 11% Fat: 49%	Hydrolysed formula (55% MCT fat).
12	NICCD	−2.54	−4.51	Eats sweet puddings.	Casein hydrolysed formula with MCT.	CHO: 43% Protein: 12% Fat: 45%	Hydrolysed formula (55% MCT fat).
13	FTTDCD	−0.19	−0.44	Does not eat cake or biscuits; rarely eats rice, pasta and bread. Prefers high protein foods (yoghurt, milk, meat, eggs).	Low sugar, high protein/ high fat diet.	CHO: 47% Protein: 19% Fat: 38%	Not prescribed.
14 (Family 3)	FTTDCD	0.69	0.91	Dislikes sweets. Avoids CHO and prefers high protein foods (meat/cheese/milk).	Normal diet.	CHO: 28% Protein: 26% Fat: 46%	Not prescribed.
15 (Family 3)	Family screening	−0.16	−0.08	Dislikes sweets. Avoids vegetables and fruits. Craves high protein foods, particularly yoghurt. Likes cream.	Low sugar, high protein, high fat diet.	CHO: 32% Protein: 20% Fat: 48%	Full fat milk.
16	NICCD	−1.04	0.02	Dislikes sweets. Avoids high CHO containing foods. Prefers high protein foods (egg, meat, cheese).	Normal diet.	CHO: 43% Protein: 17% Fat: 37%	Full fat milk.
17	Family screening	−2.71	−1.70	Avoids sugary foods. Likes yoghurt.	Lactose free diet (soya milk).	CHO: 41% Protein: 15% Fat: 47%	Soya milk.
18	NICCD	−0.26	−0.22	Self-selects a high protein and high fat diet. Dislikes sweets and fizzy drinks.	Low sugar, high protein, high fat diet.	CHO: 42% Protein: 19% Fat: 38%	Full fat milk, nuts.
19 (Family 4)	Family screening	−0.89	−0.40	Likes CHO containing foods and sweets.	Normal diet.	CHO: 44% Protein: 16% Fat: 38%	Not prescribed.
20 (Family 4)	FTTDCD	−1.25	−0.53	Dislikes CHO containing foods and fruit. Likes meat.	Normal diet.	CHO: 42% Protein: 15% Fat: 43%	Not prescribed.
21	FTTDCD	−1.41	−1.74	Dislikes fruit and vegetables. Prefers chicken.	Normal diet.	CHO: 46% Protein: 17% Fat: 35%	Not prescribed.
22	NICCD	−2.69	−0.46	Dislikes vegetables. Prefers cheese.	Low sugar, high protein, high fat diet.	CHO: 40% Protein: 17% Fat: 41%	Not prescribed.
23	Family screening	−3.47	−1.04	Likes high protein foods.	Low sugar, high protein, high fat diet.	CHO: 43% Protein: 14% Fat: 43%	Not prescribed.
24	NICCD	−2.97	−1.29	Dislikes rice, potatoes and pasta but likes chicken and fish.	Low sugar, high protein, high fat diet.	NA	Full fat milk.
25	NICCD	0.22	−0.43	Likes meat.	Low sugar, high protein, high fat diet.	NA	Full fat milk.
26 (Family 5)	Family screening	−2.29	−2.12	Avoids many CHO containing food, fruit and vegetables. Prefers high protein foods.	Normal diet.	CHO: 41% Protein: 22% Fat: 36%	Full fat milk.
27 (Family 5)	Family screening	−0.64	−0.15	No food aversions. Likes dairy products.	Normal diet.	CHO: 44% Protein: 20% Fat: 36%	Full fat milk.
28 (Family 5)	NICCD	−0.82	−0.39	No food aversions. Likes chicken.	Normal diet.	NA	Full fat milk.
29 (Family 5)	Family screening	−0.99	−1.38	Likes chicken and cheese. Does not like dairy products.	Normal diet.	CHO: 49% Protein: 13% Fat: 38%	Full fat milk.
30 (Family 5)	Family screening	−1.43	−0.91	No preferences.	Normal diet.	CHO: 48% Protein: 20% Fat: 32%	Full fat milk.
31 (Family 5)	Family screening	−0.89	−0.89	Dislikes sweet foods. Poor tolerance to dairy foods.	Normal diet.	CHO: 31% Protein: 23% Fat: 42%	Full fat milk.
32	FTTDCD	−0.57	−0.96	Dislikes sugary foods. Prefers savoury foods.	Normal diet.	CHO: 37% Protein: 23% Fat: 38%	Soya milk, nuts.

Abbreviations: MCT—medium chain triglyceride; LCT—long chain triglyceride; CHO—Carbohydrates; NICCD—neonatal intrahepatic cholestasis; FTTDCD—failure to thrive and dyslipidaemia caused by citrin deficiency; NA—Not available.

**Table 6:** Median percentage of energy contribution from protein, fat and CHO calculated from dietary assessments.

	Carbohydrates Median % [Range]	Protein Median % [Range]	Fat Median % [Range]
**Total (*n* = 27)**	42% [18–49]	19% [11–27]	40% [32–55]
**NICCD (*n* = 9)**	42% [18–47]	18% [11–27]	41% [33–55]
**FTTDCD (*n* = 7)**	42% [28–47]	19% [15–26]	38% [35–51]
**Family screening (*n* = 11)**	42% [28–49]	20% [13–23]	40% [32–53]

Abbreviations: NICCD—neonatal intrahepatic cholestasis; FTTDCD—failure to thrive and dyslipidaemia caused by citrin deficiency.

**Table 7:** Median percentage of energy contribution from carbohydrate, protein and fat of patients on prescribed compared to no ‘formal’ diet therapy.

	Carbohydrate	Protein	Fat
**Normal diet (*n* = 16)**	43% [28–49]	19% [13–26]	38% [32–51]
**Prescribed low-carbohydrate, high-protein, high fat diet** **(*n* = 11)**	40% [18–47]	19% [11–27]	45% [38–55]
